# Lifestyle and work-related correlates of psychosocial health among Australian teachers: a cross-sectional study

**DOI:** 10.1007/s10389-023-01874-9

**Published:** 2023-03-22

**Authors:** Lucy Corbett, Adrian Bauman, Louisa R. Peralta, Anthony D. Okely, Philayrath Phongsavan

**Affiliations:** 1 Sydney School of Public Health and The Charles Perkins Centre, The University of Sydney, Sydney, New South Wales Australia; 2 Sydney School of Education and Social Work, The University of Sydney, Sydney, New South Wales Australia; 3 Early Start, Faculty of Social Sciences, University of Wollongong, Wollongong, New South Wales Australia; 4 Illawarra Health and Medical Research Institute, Wollongong, New South Wales Australia

**Keywords:** School teachers, Health behaviour, Exercise, Diet, Burnout professional, Psychological distress

## Abstract

**Aim:**

This study examined the psychosocial (psychological distress, job-specific wellbeing, burnout) health of a large sample of teachers in New South Wales (NSW), Australia, specifically the association between psychosocial health, work-related factors, and lifestyle behaviours.

**Subject & methods:**

An online survey collected lifestyle behaviours, work-related factors, and socio-demographics from primary and secondary school teachers in NSW from February to October 2021. Associations between work-related factors, lifestyle behaviours, and psychosocial health were modelled using logistic regression in R and adjusted for gender, age, and geographic location.

**Results:**

In our sample (*n* = 1136), 75% were women and 28% of the sample worked in rural or remote areas. Women reported higher levels of psychological distress (51%), compared with men (42%), and over 30% of teachers reported high levels of burnout. Teachers who engaged in three or more positive health-related behaviours had lower odds of psychological distress and burnout as well as higher odds of job-specific wellbeing. Multiple work-related factors such as hours worked, teaching load, teaching experience, teacher type, and teacher role were associated with one or more aspects of psychosocial health after adjusting for socio-demographic variables.

**Conclusion:**

More is needed to support the psychosocial health of teachers in NSW. Future lifestyle programs for this population should include psychosocial outcomes to further explore the relationship between teachers’ health-related behaviour and their psychosocial health.

**Supplementary information:**

The online version contains supplementary material available at 10.1007/s10389-023-01874-9.

## Introduction

Teaching is an increasingly demanding profession, as teachers face many intrapersonal, interpersonal, organisational, and administrative challenges which contribute to high levels of stress (Thomson and Hillman 2020). The stressful nature of teaching detrimentally impacts teachers’ health with teachers having poorer physical and psychosocial health compared with the general population (Bogaert et al. 2014; Temam et al. 2022). Several studies have found teachers to have higher levels of psychological distress and non-specific symptoms of stress, anxiety, and depression than other professionals from the general population (Titheradge et al. 2019). If left unmanaged, prolonged exposure to occupational stress can lead to burnout which is characterised by feelings of exhaustion, cynicism towards one’s job, and reduced self-efficacy for teaching (World Health Organisation 2019). In Australia, stress leave taken by teachers is increasing and during 2016–17, 8% of all serious work-related mental health claims were made by teachers (Safe Work Australia 2020). In comparison, 6% of work-related mental health claims were made by health and welfare support workers and 9% were made by defence force workers, police, and firefighters combined (Safe Work Australia 2020). Whilst there is no robust Australian data on teacher burnout, global levels of burnout have been estimated to be approximately 25–40% (García-Carmona et al. 2019; Thomson and Hillman 2020). Evidence suggests that burnout contributes to the universally high attrition levels within the teaching profession (Chambers Mack et al. 2019). Given the importance of teacher retention for education system stability and the development of an experienced workforce, teacher psychosocial wellbeing is an important issue to identify potential areas for further support.

Teachers’ workload is widely recognised as being associated with their psychosocial health (Jomuad et al. 2021; Kreuzfeld et al. 2022). However, other work-related factors may impact teachers’ psychosocial health. Qualitative research suggests that having an ongoing contract can influence teachers’ job-specific wellbeing; a concept which relates to aspects of working life, from self-efficacy to safety and job security (Marent et al. 2020). Additionally, research into teacher wellbeing has focused on early-career teachers due to the high rates of attrition in this population (McCallum et al. 2017). More years of teaching experience was associated with higher levels of wellbeing (Van Petegem et al. 2005). In addition to ongoing workplace stressors, COVID-19 has posed additional challenges to teachers as they navigated at-home learning and teaching. These changes may have exacerbated teachers’ psychosocial health (Lacomba-Trejo et al. 2022; Ozamiz-Etxebarria et al. 2021).

Health-promoting behaviours such as regular physical activity, balanced nutrition, and adequate sleep are important for reducing the risk of chronic disease (Lee et al. 2012) and maintaining psychosocial wellbeing (Dale et al. 2014). Evidence indicates that physical activity interventions can reduce stress and depression and have a positive effect on work-related outcomes such as job satisfaction, self-efficacy, and absenteeism among workers (Barr-Anderson et al. 2011; Proper and van Oostrom 2019). Other lifestyle behaviours such as healthy nutrition (Young et al. 2019) and sufficient sleep (Konjarski et al. 2018) are associated with mood and can reduce stress levels in the general population. A recent scoping review found that lifestyle interventions have the potential to improve mental wellbeing of teachers, but few quality studies exist, which highlights that current evidence is inconclusive (Corbett et al. 2022). Recognising the importance of both work-related factors and health-related behaviours, this study examined work-related and lifestyle factors associated with psychosocial wellbeing among teachers in New South Wales (NSW), Australia. We hypothesized that work-related factors and health behaviours will be associated with the psychological distress, wellbeing and burnout of teachers.

## Method

This study followed the strengthening the reporting of observational studies in epidemiology (STROBE) statement reporting guidelines (von Elm et al. 2007). The research was approved by the Human Research Ethics Committee, The University of Sydney (Protocol No. 202/325).

### Study design

This cross-sectional study used an online survey to collect information on socio-demographic characteristics, lifestyle behaviours, and psychosocial and work-related factors from primary and secondary school teachers living in the state of NSW, Australia. This included schools from Government, Independent, and Catholic education sectors which respectively make up 69%, 12% and 19% of schools in NSW. A sample of schools was generated using stratified single-stage cluster sampling design. Schools were sampled with probabilities proportional to the number of teaching staff from the list of all schools in NSW. The sample of schools to be sampled was calculated to be 220, and a list of 500 schools was generated. Due to the challenges related to COVID-19 and the impact the pandemic had on schools, recruitment through random sampling was unattainable and convenience sampling was used. The revised sample size calculation used an expected proportion = 0.5, desired precision = 0.05, and α = 0.05, and provided a target sample size of 385 teachers.

### Recruitment and participants

The survey opened on 4 February 2021 and closed on 12 October 2021. In Australia, there were rapid changes in restrictions due to COVID-19 during the survey period. Between February 4 and June 24, NSW was not heavily impacted by COVID-19, with only a few restrictions in place. From June 25 to October 11, there were COVID-19 restrictions in place across NSW which varied in intensity depending on region (e.g., Sydney metropolitan was in lockdown from 25 June whilst NSW regional areas were not in lockdown until 14 August).

Three methods of recruitment were used:

1. Principals of 500 randomly selected schools were emailed information about the study and invited to forward the survey link to teaching staff at their school. Publicly available school email addresses were used.
2. Professional associations with ties to NSW teachers promoted the survey through internal communication and social media.
3. Targeted Facebook advertising was also used to reach teachers in NSW.

Eligible participants were those who consented to participate and were currently employed as a primary or secondary teacher at a school in NSW.

### Measures

Consenting participants completed a self-administered questionnaire through an online and secured digital platform (Qualtrics). The full questionnaire can be found in Supplementary S1. Briefly, the questionnaire included:

#### Demographics

Participants were asked their gender, age, and geographical location. Geographical location was reported by identifying their school’s region on a map and describing their school's location as urban (densely populated major city), suburban (lower density outskirts of a metropolitan area), rural, or remote. At the time of the survey, COVID-19 outbreaks were occurring in NSW. An adapted question from Gentili and colleagues was included to measure their perceived risk of COVID-19 on a four-point Likert scale (Gentili et al. 2020).

#### Work-related factors

Participants were asked: the year that they were first employed as a teacher (teaching experience), which educational sector their current school resided in (school sector), whether they had a full-time (1.0 full-time equivalent) or part-time (< 1.0 full-time equivalent) load (teaching load), whether their employment contract was permanent, fixed-term or casual (contract type), whether they taught primary or secondary school (teacher type), and whether they had any leadership positions at school (e.g., head of department, year co-ordinator, deputy principal, principal). A single item question adapted from the Australian Teacher Workforce Data (ATWD) Survey 2020 was used to measure hours worked in an average week (Australian Institute for Teaching and School Leadership 2021).

#### Lifestyle behaviours

##### Physical activity

A validated single item question (Milton et al. 2011) was used to measure physical activity in the previous week. Meeting physical activity guidelines was classified as participating in at least 30 minutes of physical activity ≥ 5 days/week, which is in line with the Australian Physical Activity Guidelines for adults (Australian Department of Health 2017).

##### Fruit, vegetable, and sugar-sweetened beverage (SSB) consumption

Daily fruit, vegetable, and SSB consumption was measured using questions from the NSW Population Health Survey 2019, a serial cross-sectional population-wide telephone-based survey of residents aged 16 years and older living in NSW (Centre for Epidemiology and Evidence 2019). These questions have reasonably good validity when compared with 24-hour recall (Flood and Webb 2005). In line with the Australian Dietary Guidelines, consuming ≥ 2 serves of fruit/day and ≥ 5 serves of vegetables per day were classified as meeting guidelines (NHMRC 2013). SSB intake was categorised into daily or less than daily consumption to distinguish between regular and occasional consumers (National Health and Medical Research Council 2013).

##### Current smokers

Two previously validated (Barr et al. 2008) questions from the NSW Population Health Survey (Centre for Epidemiology and Evidence 2019) were used to measure cigarette and e-cigarette smoking status. Those who smoked either cigarettes or e-cigarettes daily or occasionally were classified as current smokers.

##### Alcohol consumption

Validated (Barr et al. 2008) questions from the NSW Population Health Survey (Centre for Epidemiology and Evidence 2019) were used to measure alcohol consumption. To measure short-term risky drinking, participants were asked whether they had consumed more than four standard drinks on one occasion in the last 4 weeks. Long-term risk of drinking was measured by asking participants the frequency of alcohol consumption and the average number of standard drinks consumed. As reflected by the 2020 Australian national guidelines (National Health and Medical Research Council 2020), those who consumed more than ten standard drinks in a week or more than four standard drinks on one occasion were classified as not meeting guidelines.

##### Body mass index (BMI)

BMI was classified into underweight/healthy weight (BMI <25 kg/m^2^) and overweight/obesity (BMI ≥ 25kg/m^2^) using the calculated BMI (kg/m^2^) from self-reported height (cm) and weight (kg) measurements.

##### Sleep

Sleep duration was assessed by asking participants what time they usually go to bed and what time they usually wake up (Adams et al. 2016). The National Sleep Foundation’s guidelines state that sleeping for 7–9 hours/night on average (Hirshkowitz et al. 2015) was classified as meeting sleep guidelines for adults.

Sleep quality was assessed using a single-item sleep quality scale where participants were asked to rate their sleep quality on a 5-point Likert scale from poor to excellent (Snyder et al. 2018). The validation details of the sleep quality scale are published elsewhere (Snyder et al. 2018).

#### Psychosocial wellbeing

##### Psychological distress

Psychological distress was measured using the validated ten-item Kessler Psychological Distress Scale (K10) (Kessler et al. 2002) that measures anxiety, depression, agitation, and psychological fatigue in the most recent 4-week period. Participants with a K10 score of 22 or above were classified as having high or very high levels of psychological distress (Centre for Epidemiology and Evidence 2019).

##### Job-specific wellbeing

Teachers’ job-specific wellbeing was measured using the validated eight-item self-report Teacher Subjective Wellbeing Questionnaire (Renshaw et al. 2015). A score less than 20 was categorised as almost never/sometimes having wellbeing and a score of 20 or more was categorised as often/almost always having wellbeing.

##### Teacher burnout

A 21-item Teacher Burnout Scale (Seidman and Zager 1987) was used to measure teacher burnout. The validity of the Teacher Burnout Scale has been reported elsewhere (Seidman and Zager 1987). Participants scores were divided into tertiles, and respondents in the highest tertile were classified as ‘higher levels of burnout’ compared with participants in the low and middle tertiles.

### Statistical analysis

To examine the combined effects of multiple healthy behaviours, we constructed a healthy lifestyle index by summing those who met the guidelines for physical activity, fruit, vegetable, and SSB consumption, sleep, and smoking behaviour into a single index. This was based on a method developed by Ding and colleagues (Ding et al. 2014). Due to missing data, alcohol consumption was excluded (i.e., a number of teachers in this sample preferred not to answer questions pertaining to alcohol consumption). Using Ding and colleagues’ method (Ding et al. 2014), healthy behaviour (e.g., meeting sleep guidelines, not smoking) was coded 1 so that when the lifestyle behaviours were combined a higher score indicated that the individual had healthier behaviour. Individuals with scores of 3 or less were classified as ‘unhealthy’ while those with scores of 4 or higher were deemed ‘healthy’. Despite the missing alcohol data, analyses with alcohol included in the index can be found in Supplementary S2.

**Fig. 1:**
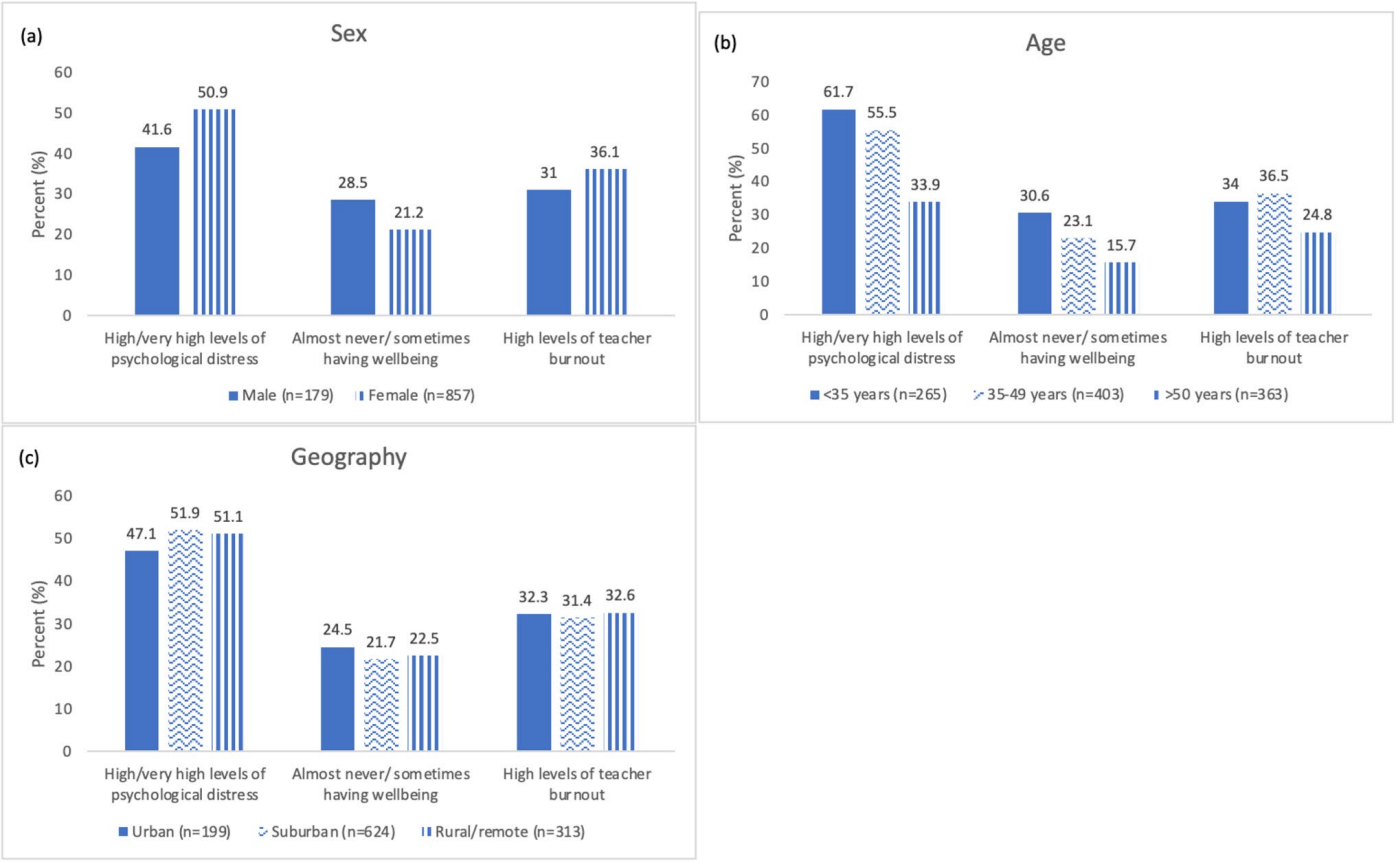
Teachers’ psychosocial factors according to (**a**) gender, (**b**) age and (**c**) geography

Descriptive analyses were reported by demographic characteristics (see Fig. 1). Associations between individual work-related factors and psychosocial factors as well as associations between lifestyle behaviours and psychosocial factors were modelled using logistic regression. Analyses were adjusted for gender, age, and geographical location since these demographics are known to impact psychosocial factors (Caldwell et al. 2004; Kelly et al. 2010). All analyses were conducted using R version 4.0.3 with epiR (version 2.0.48), car (version 3.1.0), and jtools (version 2.2.0) packages.

## Results

### Demographics

Of the 500 schools contacted, 13 schools participated the study giving a school response rate of 2.6%. When combined with responses from other methods of recruitment (i.e., recruited through member organisations), our survey sample consisted of 1136 teachers. Most participants were women (75%), and about half (55%) taught at schools in suburban areas. There were relatively fewer teachers aged < 35 years (23%), compared with those aged 35–49 years (35%) and > 50 years (32%). Figure 1 presents psychosocial experiences of the sample according to gender, age, and geographical location. Female teachers had higher levels of psychological distress and burnout compared with males. Teachers under 35 years old had higher levels of psychological distress, lower levels of job-specific wellbeing, and higher levels of burnout compared with teachers aged over 50 years.

### COVID-19

Teachers who perceived the risk of COVID-19 to be ‘high’ had greater odds of having high/very high levels of psychological distress (OR: 1.6, 95% CI:1.1–2.4), compared with those who perceived the risk as low. Those teachers who perceived COVID-19 to be a ‘moderate risk’ did not have higher levels of psychological distress (OR: 1.0, 95% CI: 0.8–1.3) (see Supplementary S3 for more details).

### Association between work-related factors and psychosocial wellbeing

 Table 1 shows teachers who worked 60+ hours had greater odds of high/very high levels of psychological distress (OR: 2.1, 95% CI: 1.4–3.2) and greater odds of burnout (OR: 1.6, 95% CI: 1.0–2.5). The odds of having high/very high psychological distress were 29% lower among secondary school teachers and 33% lower among part-time teachers.

**Table 1:** Associations between work-related factors and poor psychosocial outcomes adjusted for age, gender, geographic location

	*N*	High/very high levels of psychological distress		Almost never/sometimes having wellbeing		High levels of teacher burnout	
		OR (95% CI)	*P* value	OR (95% CI)	*P* value	OR (95% CI)	*P* value
Hours worked/week			< 0.001		0.056		0.017
< 40 (ref)	170						
40–49	230	0.84 (0.54–1.30)		0.59 (0.36–0.99)		0.91 (0.57–1.46)	
50–59	423	1.30 (0.87–1.92)		0.65 (0.42–1.01)		0.97 (0.64–1.47)	
60+	273	2.07 (1.35–3.19)		0.95 (0.59–1.52)		1.59 (1.02–2.47)	
Teaching load			0.013		0.079		0.946
Full time (ref)	898						
Part time	238	0.67 (0.49–0.92)		1.38 (0.97–1.96)		0.99 (0.71–1.37)	
Contract type			0.323		0.079		0.035
Permanent (ref)	839						
Fixed term/casual	297	1.16 (0.86–1.55)		1.34 (0.97–1.86)		0.72 (0.52–0.98)	
Teacher role			0.046		< 0.001		< 0.001
Teacher (ref)	840						
Leadership position (HOD, deputy, principal)	296	0.74 (0.55–0.99)		0.43 (0.29–0.65)		0.57 (0.41–0.80)	
Teacher type			0.013		0.056		0.652
Primary (ref)	479						
Secondary	563	0.71 (0.54–0.93)		0.73 (0.53–1.01)		0.94 (0.70–1.25)	
Teaching experience			0.092		0.475		<0.001
< 5 years (ref)	163						
6–15 years	340	0.90 (0.59–1.36)		0.98 (0.62–1.53)		2.44 (1.53–3.87)	
16+ years	632	0.63 (0.38–1.02)		0.76 (0.44–1.31)		2.23 (1.30–3.82)	

*HOD*, head of department

Teachers with 15 to 16 years of experience (OR: 2.4, 95% CI:1.5–3.9) and 16+ year of teaching experience had greater odds of burnout (years (OR: 2.2, 95% CI: 1.3–3.8). Those teachers on a fixed-term or casual contract were 28% less likely to have burnout. Teachers in a leadership position were 26% less likely to have psychological distress, 57% less likely to have poor job-specific wellbeing, and 45% less likely to have burnout compared with teachers not in a leadership position.

### Association between lifestyle behaviours and psychosocial wellbeing

Table 2 shows the associations between various lifestyle factors and psychosocial outcomes adjusted for age, gender, and geographic location. Teachers who consumed less than the daily recommended consumption levels of SSBs were 53% less likely to have psychological distress and 44% less likely to have poor job-specific wellbeing. Meeting alcohol guidelines was associated with lower odds of psychological distress (OR: 0.7, 95% CI:0.5–0.9).

**Table 2:** Associations between lifestyle behaviours and poor psychosocial outcomes adjusted for gender, age and geographic location

	*N*	High/very high levels of psychological distress		Almost never/sometimes having wellbeing		High levels of teacher burnout	
		OR (95% CI)	*P* value	OR (95% CI)	*P* value	OR (95% CI)	*P* value
Meeting PA guidelines			0.092		0.260		0.625
No (ref)	859						
Yes	256	0.77 (0.57–1.04)		0.81 (0.56–1.17)		0.92 (0.67–1.27)	
Meeting fruit consumption guidelines			0.156		0.361		0.353
No (ref)	656						
Yes	445	0.83 (0.64–1.07)		0.87 (0.64–1.18)		0.88 (0.67–1.15)	
Meeting vegetable consumption guidelines			0.593		0.223		0.087
No(ref)	993						
Yes	107	0.89 (0.58–1.37)		0.71 (0.41–1.25)		0.66 (0.40–1.08)	
SSB consumption			< 0.001		< 0.001		0.054
Daily (ref)	234						
Less than daily	867	0.47 (0.34– 0.65)		0.56 (0.40–0.79)		0.73 (0.53–1.00)	
Current smoker			0.171		0.912		0.767
Yes (ref)	84						
No	1016	0.72 (0.44–1.16)		1.03 (0.59–1.80)		0.93 (0.57–1.51)	
Meets alcohol guidelines			0.017		0.763		0.759
No (ref)	357						
Yes	525	0.70 (0.52–0.94)		0.95 (0.67–1.34)		1.05 (0.77–1.42)	
Meets sleep guidelines			0.029		0.224		0.018
No (ref)	402						
Yes	662	0.75 (0.57–0.97)		0.83 (0.61–1.120)		0.72 (0.55–0.95)	
Sleep quality			< 0.001		< 0.001		<0.001
Poor (ref)	226						
Fair	403	0.24 (0.16–0.35)		0.48 (0.33–0.69)		0.43 (0.31–0.61)	
Good	283	0.12 (0.08–0.19)		0.46 (0.30–0.68)		0.35 (0.24–0.500	
Very good/excellent	117	0.07 (0.04–0.12)		0.20 (0.10–0.38)		0.35 (0.21–0.58)	
BMI			0.009		0.969		0.439
Underweight/healthy weight (ref)	355						
Overweight/obesity	597	1.45 (1.10–1.91)		0.99 (0.72–1.37)		1.12 (0.84–1.49)	
Lifestyle index			< 0.001		0.002		0.007
Unhealthy	715						
Healthy	348	0.56 (0.43–0.74)		0.58 (0.41–0.82)		0.67 (0.50–0.90)	

*SSB*, sugar sweetened beverage, *BMI*, body mass index

Teachers who met the sleep guidelines were 25% less likely to have high or very high levels of psychological distress, and those who had very good or excellent sleep quality were 93% less likely to have psychological distress. Participants with overweight/obesity were 45% more likely to have high or very high levels of psychological distress. No significant associations were found between any of the psychosocial factors and smoking status, or meeting physical activity, fruit, or vegetable guidelines.

### Healthy lifestyle index and psychosocial wellbeing

The healthy lifestyle index was associated with all three psychological outcomes. On average, healthy individuals were 44% less likely to be classified as having high or very high psychological distress (*p* < 0.001), 42% less likely to have poor job-specific wellbeing (*p* < 0.01), and 33% less likely to suffer burnout (*p* < 0.01) compared with unhealthy individuals.

## Discussion

Half of female teachers and around 42% of male teachers in our sample had high or very high levels of psychological distress. In comparison, approximately 19% of women and 16% of men in the overall NSW adult population have high or very high levels of psychological distress (Centre for Epidemiology and Evidence 2019). Additionally, over 30% of teachers reported high levels of burnout, which is comparable to estimates presented in previous research (García-Carmona et al. 2019). The high levels of psychological distress and burnout may partially be explained by additional stresses caused by the COVID-19 pandemic, with current evidence suggesting that teachers reported heightened stress during this period (Lacomba-Trejo et al. 2022; Ozamiz-Etxebarria et al. 2021).

Our study found multiple work-related factors associated with teachers’ psychosocial health. Hours worked per week and teaching load were significantly associated with psychological distress, and those who worked more than 60 hours per week had higher odds of burnout. This demonstrates the negative impact limited leisure time may have on teachers’ psychosocial health (Jomuad et al. 2021; Kreuzfeld et al. 2022). Psychological distress was also associated with teacher type. Secondary school teachers in our sample tended to have lower odds of having high psychological distress than primary teachers. This finding is supported by another Australian study which found primary school teachers had higher levels of stress and burnout compared with secondary teachers (Carroll et al. 2022). In Australia, primary school teachers often work alone and need to take on many roles with their students such as temporary parent, counsellor, sports coach, disciplinarian, and first-aid officer (Hasan and Azad 2014). In addition to playing multiple roles, primary teachers may feel responsible for the intellectual, social, emotional, and physical development of their students, which contributes to stress (Hasan and Azad 2014). To date, limited studies have compared the psychosocial health of primary and secondary teachers, as most studies focus on recruiting teachers from one type of school. Thus, it is recommended that future studies examine the psychosocial health of both primary and secondary teachers.

Teachers in our sample who had 6 or more years of experience had higher odds of burnout compared with teachers with less experience. This finding was initially unexpected since previous research suggests teachers with more experience have higher levels of wellbeing (Van Petegem et al. 2005). However, since our survey commenced at the start of the school year, the early career teachers who were burnt out may have left the profession. Globally, teacher attrition is highest during the first 5 years (Weldon 2018), low during midcareer, and returns to being high as retirement approaches (Borman and Maritza Dowling 2008; Droogenbroeck and Spruyt 2014). A previous research study conducted with Australian early-career teachers found a high workload with low rewards were sources of stress and reasons for leaving the profession (Goddard and Goddard 2006). However, mid-career and older teachers who stay in the profession face sustained exposure to stress, which may lead to burnout. Additionally, more experienced teachers may feel ‘trapped’ as there are limited alternate work-related options (Gazi et al. 2015). Our results may also be partially explained by COVID-19 lockdowns. In the middle of the school year, when stress and burnout are high compared with the start of the year (von der Embse and Mankin 2021), COVID-19 lockdown measures were announced. The external influence of COVID-19 may be disproportionally stressful to more experienced teachers (Lagat 2021), as they tend to be older and thus more susceptible to the virus as well as facing additional challenges related to online teaching. Our finding reinforces that stress continues to be an issue for teachers, and attention to mitigating stress is needed at any stage of a teacher’s career.

Interestingly, teachers in our sample who held a leadership position experienced less psychological distress and burnout than classroom teachers. Previous studies have found high levels of burnout among American (DeMatthews et al. 2021) and Irish (Darmody and Smyth 2016) principals and COVID-19 exacerbated these burnout levels (DeMatthews et al. 2021). Due to the small numbers of principals in our sample, we combined principals with other teachers in leadership positions, which may partially explain our result. Those in leadership positions may have more autonomy and higher decision latitude compared with teachers not in leadership positions. High job strain and less autonomy have been shown to increase employee stress (Marmot 2004); thus, teachers not in leadership positions may be more prone to stress. Higher social capital may be another possible explanation for our finding. Previous findings have presented higher levels of social capital among school principals, with those school principals also reporting higher levels of wellbeing (Beausaert et al. 2021). Teachers who are in leadership positions have higher levels of job-specific wellbeing. Principals and other school leaders may have higher levels of job autonomy, which can increase feelings of efficacy and school connectedness (Federici 2013). Feelings of efficacy and school connectedness have an inverse relationship with stress in teachers (von der Embse and Mankin 2021). Therefore, by having higher levels of school connectedness and efficacy due to more job autonomy, these factors may have played a mediating role, lowering the psychological distress and burnout among principals and school leaders.

Teachers who engaged in three or more healthy lifestyle behaviours had less psychological distress or burnout and more job-specific well-being. This finding is supported by a review which found lifestyle interventions may have a variety of positive psychosocial impacts on teachers, including improved mental well-being, anxiety, stress, relationships with colleagues, and job satisfaction (Corbett et al. 2022). However, further evidence from lifestyle interventions with teachers is needed to understand the direction of association and the extent to which improving lifestyle behaviours influences their psychosocial health (Corbett et al. 2022). Further evidence on the positive impact of lifestyle behaviours on psychosocial health can be found in research among other workplaces. Reviews of physical activity interventions among office workers (Abdin et al. 2018) and healthcare workers (Bischoff et al. 2019) have found increasing physical activity improved stress and well-being outcomes. Stress and well-being can also be influenced and improved by other healthy lifestyle behaviours such as good nutrition (Young et al. 2019) and adequate sleep (Konjarski et al. 2018). Since lifestyle behaviour change interventions benefit the physical health of participants and can prevent chronic disease (Dietz et al. 2016), we recommend further lifestyle interventions be conducted with teachers. These interventions should also measure and report on psychosocial outcomes, to contribute to evidence in this area.

### Limitations

This was a cross-sectional study, which was appropriate for providing a snapshot of the psychosocial health of NSW teachers. However, these data do not provide evidence of a causal relationship between healthy lifestyle behaviour and subsequent psychosocial health. Future studies using longitudinal or intervention study designs should be conducted to explore the temporal relationship between teachers’ lifestyle behaviour and their psychosocial health.

Due to their low cost and ease of administration, self-report questionnaires are useful for population surveillance and large cohort studies. However, weaknesses of using self-reported data include the introduction of social desirability bias and reduced validity (Kelly et al. 2016; Krumpal 2011). Whilst pre-validated measures were used in this study in attempt to reduce the weaknesses associated with self-report measures, it is important to acknowledge these limitations.

Initially, random sampling was going to be used to generate a representative sample of NSW teachers. However, due to the enormous impact COVID-19 had on the ability to conduct research in schools, the response rate using this method was low, and convenience sampling methods were used to increase the sample size. Whilst different channels were used to recruit teachers, convenience sampling may have introduced bias (Alkassim et al. 2016). In Australia especially, future studies utilising representative methods are needed to examine the lifestyle behaviours and psychosocial health of teachers.

## Conclusion

Teachers had high levels of psychological distress and burnout, suggesting more needs to be done to improve the psychosocial health of teachers in NSW. Findings from this study reinforce and extend existing literature on teachers' psychosocial health, including the role of work-related factors on individual health and wellbeing. Teachers who engaged with multiple health-related behaviours were less likely to have psychological distress and burnout and more likely to have job-specific wellbeing compared with teachers who were less healthy. These data suggest the need for interventions to promote healthy lifestyles with this population group, and to report psychosocial as well as healthy lifestyle outcomes.

## Supplementary information

ESM 1 (DOC 1065 kb)

ESM 2 (DOC 29 kb)

ESM 3 (DOC 29 kb)

## Data Availability

The data that support the findings of this study are available on reasonable request from the corresponding author. The data are not publicly available due to privacy or ethical restrictions.

## Code availability

The code used for analysis in the present study is not publicly available but is available from the corresponding author on reasonable request.
