# Supplementary material for: Lifestyle and work-related correlates of psychosocial health among Australian teachers: a cross-sectional study
Source: Z Gesundh Wiss. 2023 Mar 22:1–11. Online ahead of print. doi: 10.1007/s10389-023-01874-9 (PMC10031687; doi:10.1007/s10389-023-01874-9)
Supplement: Supplementary file 2 — (DOC 29 kb) [file 10389_2023_1874_MOESM2_ESM.doc]

Supplementary Material S2. Sensitivity analysis

Supplementary Table 1: Association between healthy lifestyle index (including alcohol consumption) and poor psychosocial outcomes adjusted for gender, age and geographic location

|  | N | High/very high levels of psychological distress | | Almost never/sometimes having wellbeing | | High levels of teacher burnout | |
| --- | --- | --- | --- | --- | --- | --- | --- |
|  |  | OR(95% CI) | p | OR(95% CI) | p | OR(95% CI) | p |
| **Lifestyle index**  Unhealthy (ref)  Healthy | 406  451 | 0.65(0.49- 0.87) | 0.004 | 0.68(0.48- 0.96) | 0.025 | 0.86(0.64-1.16) | 0.3 |
