# Supplementary material for: Lifestyle and work-related correlates of psychosocial health among Australian teachers: a cross-sectional study
Source: Z Gesundh Wiss. 2023 Mar 22:1–11. Online ahead of print. doi: 10.1007/s10389-023-01874-9 (PMC10031687; doi:10.1007/s10389-023-01874-9)
Supplement: Supplementary file 3 — (DOC 29 kb) [file 10389_2023_1874_MOESM3_ESM.doc]

Supplementary S3

Supplementary Table 2: Associations between perceived risk of COVID-19 and poor psychosocial outcomes adjusted for age, gender, geographic location

|  | N | High/very high levels of psychological distress | | Almost never/sometimes having wellbeing | | High levels of teacher burnout | |
| --- | --- | --- | --- | --- | --- | --- | --- |
|  |  | OR(95% CI) | p value | OR(95% CI) | p value | OR(95% CI) | p value |
| **Perceived risk of COVID-19**  No/low risk  Moderate risk  High risk | 327  427  193 | 1.03(0.76-1.41)  1.72(1.16-2.54) | 0.01 | 1.14(0.80-1.64)  1.04(0.66-1.63) | 0.75 | 1.17(0.84-1.63)  1.56(1.05- 2.33) | 0.09 |
